# Unilateral intravitreal dexamethasone implant for diabetic macular edema: effect in the contralateral eye

**DOI:** 10.5935/0004-2749.20200013

**Published:** 2020

**Authors:** Alicia Pareja-Ríos, Sergio Bonaque-González

**Affiliations:** 1 Department of Ophthalmology, University Hospital of the Canary Islands, Santa Cruz de Tenerife, Spain; 2 Wooptix S.L, Santa Cruz de Tenerife, Spain

**Keywords:** Dexamethasone/administration & dosage, Macular edema, Intravitreal injections, Diabetic retinopathy, Diabetes mellitus, Humans, Case report, Dexametasona/administração & dosagem, Edema macular, Injeções intravítreas, Retinopatia diabética, Diabetes mellitus, Humanos, Relatos de casos

## Abstract

We describe three patients who had previous heart diseases and nonproliferative diabetic retinopathy with clinically significant diabetic macular edema. They underwent unilateral dexamethasone intravitreal implantation. Without ophthalmological treatment in the fellow eye, patients showed marked bilateral improvement in best-corrected visual acuity, optical coherence images, and macular thickness values. These findings provide evidence of the bilateral effect of dexamethasone intravitreal implantation, which may be clinically useful in patients for whom the systemic effects of the drug may affect their general health.

## Abstract (pt)

Descrevemos três pacientes que tiveram doenças cardíacas prévias e retinopatia diabética não proliferativa com edema macular diabético clinicamente significativo. Eles foram submetidos a implante intravítreo de dexametasona unilateral. Sem tratamento oftalmológico no olho contralateral, os pacien tes apresentaram uma melhora bilateral significativa na melhor acuidade visual corrigida, nas imagens de coerência óptica e nos valores da espessura macular. Esses achados fornecem evidências sobre o efeito bilateral do implante intravítreo de dexametasona, que pode ser clinicamente útil em pacientes para os quais os efeitos sistêmicos da droga possam afetar a saúde geral do paciente.

## INTRODUCTION

The sustained-delivery 0.7 mg dexamethasone (DEX) intravitreal implant (Ozurdex, Allergan, Inc., Irvine, CA, USA) is an intravitreal corticosteroid that provides controlled release of dexamethasone from an inactive biodegradable polymer matrix. It has been shown to support the resolution of diabetic macular edema (DME) and to significantly improve best-corrected visual acuity (BCVA)^(1)^. These findings are especially evident during early stages of treatment^(2)^.

Treatment with the DEX implant does not affect glycated hemoglobin (HbA1c) or renal function (creatinine) but produces a slight increase in the lipid profile of patients with diabetes for up to 15 months after treatment^(3)^. Because this increase is greater in patients with bilateral DEX implant injections^(3)^, it may be useful to explore the effect of a single unilateral injection on the fellow eye.

## CASE REPORT

Three patients with DME were referred to the University Hospital of the Canary Islands for evaluation. All received treatment with unilateral DEX implantation.

**Figure 1:**
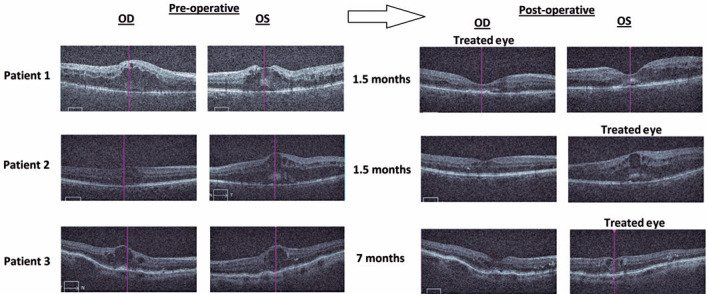
Optical coherence images of both eyes before and after unilateral dexamethasone intravitreal implantation.

**Figure 2:**
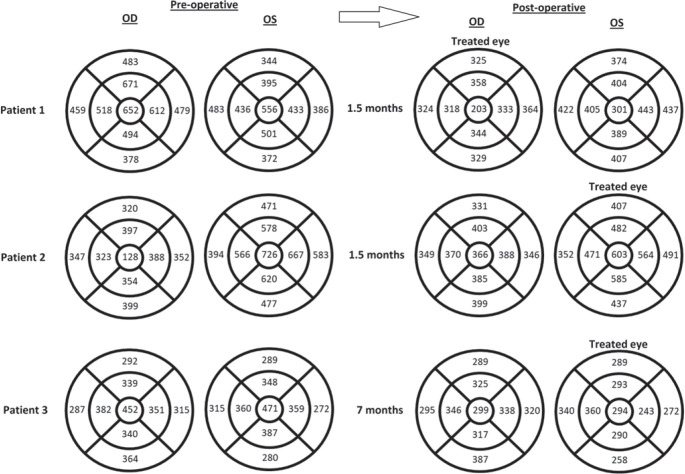
Macular thickness in microns of both eyes before and after unilateral dexamethasone intravitreal implantation.

Patient 1 was a 66-year-old woman with a 17-year history of type 2 diabetes (DM2). Her medical history included irregular glycemic control, obesity with a body mass index (BMI) of 36.1, arterial hypertension, and three coronary bypass surgeries. The patient was taking metformin, insulin and acenocoumarin. Ophthalmic examination revealed bilateral cataracts and severe nonproliferative diabetic retinopathy (NPDR) with clinically significant DME. Her BCVA was 20/400 in the right eye and 20/50 in the left eye. DEX implantation was indicated unilaterally in the right eye. Figures 1 and 2 show optical coherence images (OCT) and macular thickness before and 1.5 months after treatment, when BCVA improved to 20/50 in the right eye and 20/40 in the left eye. Her HbA1c levels were 8.2% and 8.1% at 3 months before and 1 month after DEX implantation, respectively.

Patient 2 was a 68-year-old man with a 22-year history of DM2. His medical history included regular glycemic control, arterial hypertension under treatment, obesity with a BMI of 35, dyslipidemia with statins, cardiomyopathy with previous acute myocardial infarction and coronary stents, and stopped smoking 10 years prior to the case. The patient was taking metformin. Ophthalmic examination revealed bilateral cataracts and severe NPDR with clinically significant DME. His BCVA was 20/40 in the right eye and 20/100 in the left eye. DEX implantation was indicated unilaterally in the left eye. Figures 1 and 2 show OCT images and macular thickness before and 1.5 months after treatment, when BCVA improved to 20/29 in the right eye and 20/67 in the left eye. His HbA1c levels were 7.5% and 7.7% at 5 months before and 3 months after DEX implantation, respectively.

Patient 3 was an 83-year-old woman with a 35-year history of DM2. Her medical history included regular glycemic control, osteoporosis, arterial hypertension, obesity with a BMI of 31, hypercholesterolemic dyslipidemia, cardiomyopathy with previous acute myocardial infarction, and coronary angioplasty. The patient was taking insulin, acetylsalicylic acid, and nepafenac. Ophthalmic examination showed a recent bilateral cataract surgery performed 6 months prior to this case, as well as moderate NPDR with clinically significant DME. Her BCVA was 20/100 in the right eye and 20/67 in the left eye. DEX implantation was indicated unilaterally in the left eye. Figures 1 and 2 show OCT images and macular thickness before and 7 months after treatment, when BCVA improved to 20/40 in the right eye and 20/33 in the left eye. Her HbA1c levels were 8.9% and 8.4% at 6 months before and 6 months after DEX implantation, respectively.

## DISCUSSION

Other studies have described the effects of intravitreal preparations, such as bevacizumab, ranibizumab, and triamcinolone, on DME in the contralateral eye^(4^,^5)^. In the case of DEX implants, although the effects on the contralateral eye have been shown in patients with nonin fectious uveitis^(6^,^7)^, there has only been a single report on the positive effect of DEX implantation in the contralateral eye in a patient with DME^(8)^. In the present report, we have described this finding in three additional patients, which increases the clinical evidence supporting the potential bilateral benefit of unilateral treatment with DEX implantation. Specifically, we have shown a reduction in macular thickness and enhancement in BCVA in the contralateral eye, as shown in figures 1 and 2. This effect could be useful in the treatment of some patients as the possible complications of an additional intravitreal injection are avoided. Additionally, it has been shown that, compared with unilateral treatment, bi lateral DEX implantation produces more marked increases in low-density lipoprotein cholesterol^(3)^. The reported bilateral effect can be convenient for patients who require such control, including those with a recent history (<6 months) of acute myocardial infarction.

It is unclear why DEX implantation affects the contralateral eye. It has been suggested that corticosteroid molecules may escape into the systemic circulation and subsequently reach the contralateral eye^(8)^. However, further studies are warranted to elucidate the underlying mechanism.
